# Content validity assessment and modification of the FACE-Q craniofacial module for retinoblastoma survivors

**DOI:** 10.1371/journal.pone.0339657

**Published:** 2026-03-26

**Authors:** Farheen Khan, Roxanne Noronha, Ivana Ristevski, Michelle Prunier, Genevieve Savoie, Andra Striowski, Ashwin C. Mallipatna, Karen W.Y. Wong-Riff, Asim Ali, Helen Dimaras

**Affiliations:** 1 Department of Ophthalmology and Vision Sciences, The Hospital for Sick Children, Toronto, Ontario, Canada; 2 University of Toronto, Institute of Medical Sciences, Toronto, Ontario, Canada; 3 Canadian Retinoblastoma Research Advisory Board, Toronto, Ontario, Canada; 4 Canadian Retinoblastoma Society, Ottawa, Ontario, Canada; 5 Striowski Prosthetic Eyes, Toronto, Ontario, Canada; 6 Department of Ophthalmology and Vision Sciences, University of Toronto, Toronto, Ontario, Canada; 7 Division of Plastic and Reconstructive Surgery, Department of Surgery, The Hospital for Sick Children, Toronto, Ontario, Canada; 8 Division of Plastic and Reconstructive Surgery, Department of Surgery, University of Toronto, Toronto, Ontario, Canada; 9 Child Health Evaluative Sciences Program, SickKids Research Institute, Toronto, Ontario, Canada; 10 The Centre for Global Child Health, SickKids Research Institute, Toronto, Ontario, Canada; 11 Division of Clinical Public Health, Dalla Lana School of Public Health, University of Toronto, Toronto, Ontario, Canada; Touro University California College of Pharmacy, UNITED STATES OF AMERICA

## Abstract

**Purpose:**

This study aimed to assess and modify the content of the FACE-Q Craniofacial Module, an appearance-based patient-reported outcome measure to address the lack of validated measures for use in retinoblastoma survivors.

**Methods:**

This cross-sectional qualitative content validity study was conducted at the Hospital for Sick Children. Through purposive sampling, survivors (8 years old or older) and parents of ineligible survivors participated in cognitive debriefing interviews from February 2022 to February 2024. Participants provided suggestions regarding comprehensibility, comprehensiveness, and relevance of the FACE-Q Craniofacial Module to the retinoblastoma experience. Interviews continued until saturation. The suggestions were presented to a panel of clinicians, scientists, and patient advocates to make iterative modifications.

**Results:**

Survivor participants (n = 14) and parent participants (n = 15) enrolled to the study. The mean age of participants was 20.3 and 40 years for survivors and parents respectively. The FACE-Q Craniofacial Module was modified with the revision of ten items, addition of eleven items, and removal of three items, with the Social Function (n = 7) and Appearance Distress (n = 5) scales requiring the most modifications. A novel scale of 17 items was also developed to assess the appearance of survivors who wear a prosthetic eye with input from 10 patients and 6 parents.

**Conclusions:**

The FACE-Q Craniofacial Module may be suitable to adapt for retinoblastoma survivors. The modifications resulting from this study ensure the tool comprehensively captures retinoblastoma-specific concerns, facilitating holistic and patient-centred clinical assessment and research. The next step in the adaptation of the FACE-Q Craniofacial Module is to psychometrically validate the modified version to ascertain its reliability and validity.

## Introduction

Retinoblastoma is the most common pediatric intraocular cancer, typically diagnosed before five years of age [1–3]. It is often caused by a pathogenic variant of the *RB1* gene, which can occur either as heritable germline mutations or non-heritable somatic mutations [1,2]. All bilateral cases of retinoblastoma as well as a small proportion of unilateral cases are heritable [1,2]. Retinoblastoma is curable if diagnosed early [1,2]. Small tumours can be treated via focal treatments (e.g., laser therapy, cryotherapy), while large or hard-to-treat tumours may require a combination of focal treatments and chemotherapy, plaque radiotherapy, or external beam radiation therapy. When the disease cannot be controlled through any combination of the above treatments, the eye may need to be enucleated [1,2]. Depending on the treatments received, survivors may live with life-long consequences, including poor vision, appearance-based concerns, additional cancers, and psychosocial effects [1,2,4–6].

Published qualitative research identified six domains of treatment outcomes from the perspective of retinoblastoma survivors that provide the groundwork for development of retinoblastoma-specific patient-reported outcome measures (PROMs) [4].PROMs are self-reported measurement tools that assess health and treatment outcomes as perceived by patients via standardized and validated questionnaires [7]. Currently, RetinoQuest is the only retinoblastoma-specific PROM, though it was not developed with patient input, nor validated [4,8]. The RetinoQuest covers multiple domains over 10–13 items, depending on the age-specific version [8], but does not cover any of the domains comprehensively. This limitation was indicated by the missing concepts reported by participants in a study evaluating its feasibility in clinical practice [4,8]. RetinoQuest also does not capture any appearance-based outcomes, one of the six domains of treatment outcomes identified by survivors [4].

Conversely, the FACE-Q Craniofacial Module (FACE-Q) is a rigorously developed PROM that has been psychometrically validated using Rasch Measurement Theory. It comprises 27 independent scales and checklists to evaluate appearance, facial function, health-related quality of life (HRQoL), and adverse effects of patients aged 8–29 years with noncleft craniofacial conditions [9,10]. The FACE-Q was adapted from CLEFT-Q, a PROM developed to evaluate similar outcomes in children and young adults with cleft lip and/or palate [11]. The FACE-Q aligns strongly with the guidelines from COnsensus-based Standards for the selection of health Measurement INstruments (COSMIN), international, expert-developed standards for evaluating the quality of PROMs and other health instruments [12], and includes scales and checklists specifically about the eye [9,10], supporting its suitability to adapt for evaluation of appearance-based outcomes in the retinoblastoma population.

Since FACE-Q was not developed with input from retinoblastoma survivors or patients with ocular conditions, nor is it validated for use in these patient populations, assessing its content validity is an important first step [9,10]. Content validity refers to a measurement property that evaluates whether PROM content adequately reflects the perspective of the patient population(s) of interest [13]. Content validity includes assessment of: (a) relevance of items to the outcome being measured and the patient population(s); (b) comprehensiveness of content; and (c) comprehensibility of instructions, items, and response options to the patient population(s) [13]. Additionally [14], there is no parent-proxy version of the FACE-Q for administration to caregivers of patients who are under 8 years of age or unable to self-administer the PROM [15]. Given that retinoblastoma affects young children under 8 years of age and some survivors are developmental delayed (e.g., in cases of 13q deletion) [2], a parent-proxy version would allow caregivers to evaluate outcomes on behalf of patients who are unable to self-report.

[15] As such, with input from survivors and parents, the objective of this study was to adapt the FACE-Q by assessing its content validity via cognitive debriefing interviews for use in retinoblastoma survivors, and generating new items and scales to address concepts deemed by participants as relevant but absent from the existing FACE-Q.

## Materials and methods

This cross-sectional and qualitative content validity study was approved by the Hospital for Sick Children (SickKids) Research Ethics Board (REB#1000079009) as a broader study exploring content validity of FACE-Q for use in patients with retinoblastoma, corneal anesthesia, or strabismus. The current manuscript focuses on the results of the retinoblastoma cohort.

### Study participants

#### Eligibility criteria.

As the original FACE-Q was developed for patients aged 8–29 years of age, retinoblastoma survivors at SickKids were eligible to participate if they were 8 years old or older. Parents were eligible if their child was under 8 years of age or developmentally delayed. Survivors with developmental disabilities and anyone lacking English proficiency were ineligible.

#### Recruitment and sampling strategy.

Using a purposive sampling strategy, participants were identified by reviewing the SickKids electronic medical record. Key demographic (e.g., age, sex) and clinical characteristics (e.g., disease laterality, treatment history) were monitored during recruitment, and participants were selectively approached to capture a range of perspectives and survivorship experiences to inform the content validity assessment. Between February 2022 and February 2023, eligible patients and families were first mailed letters from a member of their circle of care, then contacted by phone by a member of the research team. Written informed consent was obtained from all participants. For minors, consent was obtained from a substitute decision maker (parents or guardians), and written assent was also required.

#### Sample size.

Two rounds of interviews were conducted, resulting in working and pilot modified versions of the FACE-Q, respectively. Based on COSMIN guidelines, a minimum of seven survivor and seven parent participants were sought for each round [16]. A maximum number of participants was not set, as sampling continued until data saturation was achieved within survivor and parent groups separately (i.e., when no new relevant feedback emerged across participants in consecutive interviews)

### Data collection

#### FACE-Q scales and checklists.

Of the 27 FACE-Q scales and checklists, seven were hypothesized to be applicable to the experiences of retinoblastoma survivors and thus assessed for content validity (Table 1).

**Table 1:** FACE-Q scales and checklists adapted for retinoblastoma survivors.

Scale or Checklist	Domain	Title	Number of Items	Eye or HRQoL
Scale	Appearance	Appearance of the Eyes	9	Eye
Checklist	Function	Eye Function	7	Eye
Checklist	Adverse Effects	Eye Adverse Effects	7	Eye
Scale	HRQoL^a^	Appearance Distress	8	HRQoL
Scale	HRQoL^a^	Psychological Function	10	HRQoL
Scale	HRQoL^a^	School Function	10	HRQoL
Scale	HRQoL^a^	Social Function	10	HRQoL

^a^HRQoL = Health-Related Quality of Life

#### Intake survey.

All participants completed an intake survey, reporting their sociodemographic characteristics (i.e., sex, gender identity, language spoken at home, population groups, religion, place of residence, highest level of education completed, employment status, marital status, annual household income, number of offspring). Participants additionally reported on their or their child’s retinoblastoma history (i.e., perceived severity, laterality, date of diagnosis, treatment). While recruitment was not stratified by the sociodemographic characteristics or retinoblastoma history, we monitored diversity in the intake survey data throughout recruitment. When gaps were identified (e.g., by sex, treatment, etc.), purposive sampling strategies were used to ensure that a diverse range of lived survivorship experiences was captured in the study.

#### Cognitive debriefing interviews.

**Fig 1:**
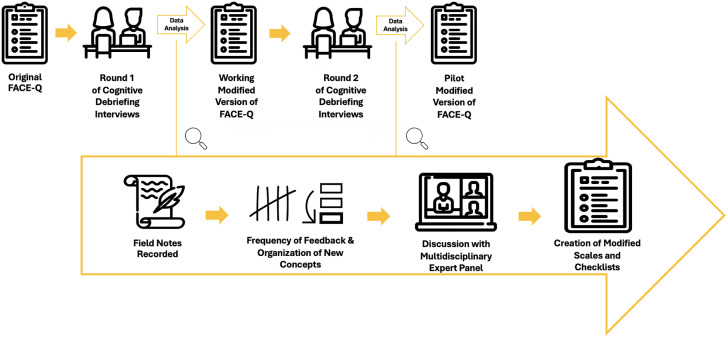
Content validity evaluation methodology for adaptation of the FACE-Q for retinoblastoma survivors. Content validity was assessed via two rounds of cognitive debriefing interviews, resulting in working and pilot modified versions of the FACE-Q, respectively. After each round, participant feedback was systemically documented and summarized for review by a multidisciplinary expert panel. Following discussion, the panel reached consensus on item modifications.

Two rounds of one-on-one cognitive debriefing interviews were conducted (Fig 1) by the first author (FK), over one year (February 2022 to February 2023), using retinoblastoma- and round-specific interview guides (S1 Text). Interviews were audio- and video-recorded. In Round 1, participants were asked to verbally answer each item in the original FACE-Q and provide insight into the (a) clarity of the instructions, (b) comprehensibility and relevance of each item, (c) comprehensiveness of each scale, and (d) appropriateness of the response options. In Round 2, participants were asked to verbally answer each item in the working modified version of the FACE-Q and provide feedback or additional clarification on (a) modifications; (b) unsettled suggestions from Round 1; and (c) the relevance of new concepts elicited in preceding Round 2 interviews.

### Data analysis

Data from the intake questionnaire was analyzed quantitatively using descriptive statistics. Qualitative data from the cognitive interviews were analyzed iteratively after each round, using a structured content analysis approach. Field notes were recorded from each interview on Microsoft Excel by FK, against probes (i.e., questions in the interview guides), on the comprehensibility and relevance of all items, comprehensiveness of each scale, and the response options, recall periods, and instructions. The type and frequency of each specific feedback were tracked on an item-by-item or scale-by-scale basis to identify suggestions for modifications (i.e., revisions, removals, and additions). Feedback from retinoblastoma participants were also cross-referenced with data from non-retinoblastoma participants in the wider study to ensure the relevance of all revised and newly added items across the patient populations. Based on recommendations in the literature [17], feedback on item comprehensibility mentioned by at least three participants, along with items deemed irrelevant by at least three participants, were systemically highlighted to discuss with a multidisciplinary expert panel. The expert panel consisted of an ophthalmologist (AM), a scientist (HD) who is a leader in retinoblastoma research, and a patient (MP) and parent (IR) advocate with lived retinoblastoma experience, to incorporate multiple stakeholder perspectives and guide consensus-based decisions regarding modifications made to the FACE-Q. The panelists were selected based on clinical,retinoblastoma research, PROM development, and lived experience expertise. In addition, novel concepts elicited by participants were organized by frequency and assessed for clinical and methodological relevance. Similar concepts were amalgamated to avoid redundancies. Given their broader relevance to retinoblastoma survivors, all relevant concepts, irrespective of their frequency, were discussed with the expert panel.

Parent and survivor suggestions were discussed simultaneously, with priority given to feedback from survivors, in alignment with the purpose of PROMs to capture the patient perspective, and to ensure consistency in content across the self-report and parent-proxy versions. While inclusion of participants with varied clinical characteristics and treatment histories was intentional to ensure comprehensive capture of diverse retinoblastoma survivorship experiences, item revisions and additions were implemented only when deemed by the panel to reflect experiences broadly applicable across demographic characteristics and treatment modalities.

After discussion with the expert panel, to ensure age-appropriate comprehension across the validated age range (8–29 years), all new and revised items were assessed using the Flesch Kincaid readability calculator to ensure a maximum reading Grade Level of 3 [18]. Items were subsequently finalized in collaboration with MP and IR.

## Results

### Study participants

The seven FACE-Q scales and checklists were reviewed by 29 participants: seven survivors and eight parents in Round 1, and seven survivors and parents each, in Round 2 (Table 2).

**Table 2:** Number of participants per scale or checklist.

		Appearance of the Eyes	Eye Function	Eye Adverse Effects	Appearance Distress	Psychological Function	School Function	Social Function
**Survivors**	**Round 1**	7	6	7	7	7	4	6
	**Round 2**	7	7	7	7	7	7	7
	**Total**	14	13	14	14	14	11	13
**Parents**	**Round 1**	8	7	8	7	7	7	7
	**Round 2**	7	7	7	7	7	7	7
	**Total**	15	14	15	14	14	14	14

While the target sample size based on COSMIN guidelines (n = 7) was met (n = 29) and saturation was achieved at the scale-level, two of the seven FACE-Q scales and checklists (Eye Function and Social Function) were evaluated by 6, rather than 7 survivor participants in Round 1 due to time constraints. Both scales were evaluated by 7 survivors in Round 2. In addition, within the survivor cohort, the School Function scale was evaluated by school-aged participants only in Round 1 (n = 4), and by all participants regardless of age (n = 7) in Round 2 (Table 2).

### Study participant demographics

The intake survey was completed by 25/29 participants (S2 Table). The average age for survivors was 19 (median: 20, range: 8–28), and 40 (median: 40, range: 33–48) for parents. 96% of survivors were treated at SickKids. Survivors predominantly identified as female (50%), white (57%), Christian (36%), and lived in large urban population centres (50%). Similarly, parents were predominantly female (53%), white (53%), Christian (40%), and lived in large urban population centres (52%). Most parents perceived their child’s condition to be moderate (53%), and survivors evenly selected between moderate and severe (29% each). There was an even breakdown of unilateral and bilateral survivor participants, whereas most of the children of the parent participants were bilateral (60%). Treatment history of survivors included enucleation, chemotherapy, focal therapy, and plaque radiotherapy.

### Modifications to FACE-Q scales and checklists

Of the 61 items in the original FACE-Q scales and checklists (Table 1), 82% required no modification. The Eye Function checklist was subdivided into two checklists, one for each eye, allowing respondents to report separately on the function of each eye. The Eye Adverse Effects checklist was subdivided into four checklists: Living Eye – Right or Left, and Prosthetic Eye – Right or Left, allowing respondents to report on adverse effects experienced by each eye based on their circumstances (S3 Table).

#### Revisions to existing items.

To improve (1) comprehensibility, (2) inclusiveness of experiences of visually impaired survivors, (3) universality and applicability to a broader range of experiences, and (4) consistency of item wording, ten items were revised (Table 3).

**Table 3:** Item revisions made to FACE-Q craniofacial.

Title of Scale	Reason for Revision	Original Item	Revised Item	Representative Quotes
**Appearance of the Eyes**	Comprehensibility	How much do you like how well your eyes suit your face?	How much do you like how well your eyes match your face?	“That's kind of a weird question in this context because, um, yeah. I don't know. It's - a little bit weird for me to think of that. It's like asking anyone “how well your eyes suit your face,” you know, like not – I don't know. I guess someone who hadn't had any procedure done on their eye, it's just a weird question to think about.” ~ RB-PT5 “I would probably rephrase that a little bit. Um, for some kids it may hold, but in our case, it just doesn't seem to match specifically. Um, and, In terms of “suit their face,” to me, honestly, that doesn't make a lot of sense, um, because again just like normal eyes, they're there, right? So it's not like she's had massive reconstruction surgery or anything like that.” ~ RB-PR8
**Appearance of the Eyes**	Consistency of item wording	How much do you like the shape of eyes (e.g., round, droopy)?	How much do you like the shape of eyes (like round, droopy)?	N/A
**Appearance Distress**	Comprehensibility	I cover up or hide how I look when I go out.	I cover up or hide how I look when I go out (like with my hair, sunglasses).	“Also, I think, like, what does it mean to “cover up and hide?” Like, how do you do that, you know? Sometimes I wish I could just like, be invisible and go outside, and just go about my day, and, but yeah, so I don't know about the wording of this question.” ~ RB-PT5. “For some reason, I - ‘cause my child, my-my son is so young, I was thinking of more like, he hides underneath his mother. Doesn't- you know, like, you know that kind of “hides,” rather than physically covers the eye, which I think, now that I read it again, was that- was what you were trying to get to for number 3.” ~ RB-PR6
**Appearance Distress**	Inclusiveness of experiences of visually impaired survivors	I get upset when people stare at me.	I don't like it when people look at or talk about my eyes.	“Um, never. But that's just in my case. Usually, um, I can't tell when people are staring at me… but it's different for everyone, so I think it's still beneficial to have.” ~ RB-PT3
**Appearance Distress**	Universality and applicability to a broader range of experiences	I avoid going out because of how I look (like to a party).	I avoid going out because of how I look.	“When I read “like to a party,” I was- I, I thought – my first, the first thought that came to my mind, it was like, teena- like older kids, okay. But like, for [child's name], I'm, I'm considering he's hanging out with uh, his friends and stuff.” ~ RB-PR1
**School Function**	Comprehensibility	I get asked to join activities and games at school.	I get asked to join activities and games at school (like in class or recess).	“No, but there also, like, really aren't like, so – like, a lot of activities and games at school, but I also don't like sports and stuff like that, so no. Like um, it means like, what does it mean by “activites?” ~ RB-PT7 “I'm not sure, like if it's relevant, because, the start of the school year they always send us a form where they ask us that whether you want your child to be, to take part, to do activities, like, they just give us a list, if you don't them, like for example, I don't want him to play the, throw ball or frisbee. So I just had mentioned those so, they always ask your child to join the activities.” ~ RB-PR3
**School Function**	Inclusiveness of experiences of visually impaired survivors	I like seeing my friends at school.	I like spending time with my friends at school.	“Not everyone can see their friends.” ~ 2RB-PT2
**Social Function**	Comprehensibility	I feel like I fit in.	I feel like I fit in with my peers.	“For me, it feels, um, like, it's common terminology for me, but it might not be for another person, like if there were language barriers or cultural barriers I think, “fit in” is not um, it might not make sense. " ~ RB-PT5 “I would have liked it to be more specific because I mean, you can look at it with peers and colleagues, or you can look at it with your friends, or with your family, so yeah.” ~ RB-PT6
**Social Function**	Inclusiveness of experiences of visually impaired survivors	It's okay when people look at my face.	It's okay when people look at or talk about my eyes.	“Yeah, I think it's relevant. For me, it's not as relevant because of my visual impairment, but I think it's good for other people.” ~ RB-PT3
**Social Function**	Universality and applicability to a broader range of experiences	I feel confident when I go out (like to a party).	I feel confident when I go out.	N/A – to keep consistent with revision in “Appearance Distress” scale.

#### Addition of items.

During the two rounds of interviews, 74 new concepts were elicited. Survivors elicited 30 new concepts (25 in Round 1; 2 in Round 2; and 3 in both Rounds), while parents elicited 61 new concepts (48 in Round 1; 8 in Round 2; and 5 in both Rounds) (S4 Table). Survivors emphasized concepts surrounding quality of life (QoL) at school or work, while parents focused on psychosocial concerns and symptoms. Upon discussion with the expert panel, 9/74 concepts aligned with the FACE-Q’s framework and were applicable to all retinoblastoma survivors regardless of treatments received.

Of the nine concepts, five were added to the HRQoL scales, and four were eye-related (S4 Table). Two additional concepts (one HRQoL, and one eye-related), originally elicited by non-retinoblastoma participants were cross-checked and identified to be relevant to the retinoblastoma experience as well (S4 Table). Therefore, a total of 11 new items were added (S3 Table).

#### Removal of items.

“The white of my eye is red” item was removed from the prosthetic-specific Eye Adverse Effects sub-checklists due to its irrelevance to wearing a prosthetic eye (S3 Table). Two additional items were removed from the Eye Function scale as they covered non-appearance-based concepts (i.e., visual function concepts).

### Modifications to the PROM components of FACE-Q

In the Appearance of the Eyes scale, an additional note, “If you wear a prosthetic eye, please answer thinking of your living eye,” was added to the instructions to enhance clarity for survivors wearing a prosthetic eye. The recall period for all scales was extended to “one month” after Round 1 based on feedback from 12 participants, then reverted to the original “one week” after Round 2. Participants (n = 12) also suggested adding a “neutral” response option, but this feedback was not incorporated. Survivors suggested this option because they felt indifferent to some of the items. Parents suggested this option because they struggled to answer several items, particularly in the HRQoL scales, from their children’s perspective. Moreover, during the think-aloud process of some interviews, it was noted that participants discussed appearance-related concerns that were not condition- or eye-specific (e.g., overall body image). Therefore, upon discussion with the expert panel, the qualifier, “With your eye condition in mind” was added to the instructions of all scales except the “Psychological Function” scale. Additional modifications included accounting for experiences with glasses in the instructions and adding a comment section to the end of each scale and checklist.

### Development of the “Appearance of the Eyes – Prosthetic” scale

Upon analysis of the 74 new concepts elicited, eight appearance-based concepts elicited were only applicable to the appearance of prosthetic eyes (S4 Table).

*“For this one, I’m like okay my eyes match each other, when um – like I guess visually, when you’re looking at, like, straight on, I think they match like relatively well, but other aspects of it, like I don’t know if there’s any questions around movement or things like that. That’s when I would say I would be lower on the scale for that because you know, if I’m looking at different directions or if I’m looking to the side, you know, that’s when, you know, it’s not going to match as well. That’s just like, tracking, so I don’t know if there’s a question like that. ~ RB-PT5*

As such, the need to develop a prosthetic-specific “Appearance of the Eyes” scale was identified. Using the eight elicited concepts, a preliminary scale was developed and evaluated through an additional round of interviews with 10 survivors and 6 parents of survivors wearing a prosthetic eye. Of the survivor participants, 6 (60%) were retinoblastoma survivors, while 4 (40%) lost their eyes due to eye trauma. All the children discussed by the parent participants (100%) were retinoblastoma survivors (S2 Table). An additional eight concepts were elicited from these interviews (S4 Table). Upon further discussion with a prosthetic-specific expert panel comprising of an ocularist (AS), ophthalmologist (AM), scientist (HD), and patient (GS) and parent (IR) advocates, a comprehensive 17-item pilot scale was developed.

## Discussion

Our study aimed to evaluate the content validity of and modify the FACE-Q to meet the unique needs of retinoblastoma survivors. A total of 82 concepts were elicited by participants, covering multiple domains (e.g., vision, QoL, etc.) in addition to appearance, highlighting the need for a retinoblastoma-specific appearance-based PROM, alongside non-appearance-based PROMs, to comprehensively capture survivors’ treatment outcomes and daily experiences.

By including multidisciplinary perspectives, our study ensured a rigorous modification process with clinical, methodological, and lived experience relevance before assessing validation and feasibility in clinical practice. By being part of a larger study, cross-checking additional suggestions elicited by non-retinoblastoma participants with retinoblastoma participants further enhanced the comprehensiveness of the scales from a broader ophthalmologic lens. Moreover, all items in the RetinoQuest, the only retinoblastoma-specific PROM developed thus far, were elicited in our study, with the exception of one regarding support services provided by companies [8]. This suggests that upon validation of the adapted FACE-Q, it may be used in conjunction with RetinoQuest for a more comprehensive evaluation of the HRQoL of retinoblastoma survivors.

In addition, as retinoblastoma survivors experience life-long consequences of the condition [4], unlike the non-retinoblastoma participants in the wider study, retinoblastoma participants in both rounds preferred extending the recall periods from a week to a month or more. However, a one-week recall was retained to align with FACE-Q’s original design and avoid recall bias, as supported by Norquist et al. [19] Similarly, a “neutral” response option was not incorporated despite suggestions from participants. This decision was based on consensus among the expert panel, and supported by prior literature demonstrating difficulty with the middle of Likert scales in young children, as well as the tendency for individuals to choose neutral options when offered, providing non-useful information [20,21].

The need to develop a novel scale evaluating the appearance of prosthetic eyes was also identified in our study. In absence of such a PROM, the FACE-Q in combination with the Derriford Appearance Scale and the Hospital Anxiety and Depression Scale was used previously to evaluate a novel artificial eye service based on patients’ experiences with their ocular prosthesis [22]. Participants in our study noted that nuanced prosthesis-specific concepts were lacking in the original FACE-Q, indicating that it is not adequately comprehensive. Another study used the UK National Artificial Eye Questionnaire (NAEQ) to understand the comfort and satisfaction of individuals wearing ocular prostheses [23]. The NAEQ consists of questions devised by prosthetics and oculoplastic experts, along with one final item for free-text testimonials from patients [23]. Unlike the NAEQ, our novel scale includes all the missing appearance-based concerns highlighted in the testimonials from the NAEQ study, and thus is more comprehensive than existing questionnaires due to the incorporation of the patient voice [23]. This includes appearance satisfaction in general (i.e., an individual’s subjective contentment), as well as nuanced satisfaction about the colour, brightness, matching with other eye, size, and shape of the prosthetic eye. Our novel scale also includes items regarding the appearance of eyelids, the fit and comfort of the prosthetic eye, feelings associated with discharge, and feelings about appearance of the prosthetic eye in photos, videos, and the mirror.

Finally, we had aimed initially to adapt the FACE-Q to develop self-report and parent-proxy-report versions. During the interviews however, particularly with HRQoL scales, we observed that while concepts were deemed to be relevant by both survivors and parent participants, parents did not know how to answer some of the items from the perspective of their child and could only discuss how they perceived their child to feel based on their own observations. Parents also elicited more concepts than survivors, especially non-appearance-related ones (S4 Table). These findings are consistent with the previous study that provided the groundwork for development of retinoblastoma-specific PROMs, in which appearance was an issue for survivors, but not caregivers [4]. In our study, most parents reported their child was not worried about appearance as they had not voiced such concerns. Our findings align with existing literature on parent-proxy versions of pediatric QoL PROMs by Ombashi et al, [24] which demonstrate that there are larger differences between parent-reported and patient-reported outcomes for social and emotional domains compared to functional domains. Moreover, as indicated by Bevans et al, [25] factors such as the quality of caregiver-patient relationships or lack of relevant discussions at home can influence how reliable and accurate a parent-proxy report is compared to a patient-report. To address these concerns, it has been recommended by developers of the CLEFT-Q to explore the option of collaborative reporting by caregivers and patients due to its several advantages such as opening up opportunities for open caregiver-patient discussions [24]. Considering that there is literature demonstrating that children do experience body image concerns and are aware of societal beauty ideals, and that pediatric patients with eye conditions in general do have appearance-based concerns [26–28], it may be more effective to explore developing a collaborative caregiver-survivor version of the FACE-Q instead of a parent-proxy version in future studies.

There are a few limitations to this study. Though there were age-specific concerns applicable to older survivors (e.g., dating, work environment, etc.) elicited for the Social Function scale, the FACE-Q for retinoblastoma survivors was adapted broadly for survivors of all ages rather than in an age-specific manner to maintain its original framework (S4 Table). However, in future studies, when developing more comprehensive HRQoL PROMs for retinoblastoma survivors, age-stratified versions should be considered. Furthermore, this study was conducted in a high-income tertiary care context. Future validation studies assessing cross-cultural validity and differential item functioning may assess whether the modified PROM functions equivalently for survivors from low- and middle-income countries. If not, additional content validity studies would be required in these health-care settings. It is also important to note that as the oldest survivor participating in this study was in their late 20s, experiences with older treatment methods, such as radiation therapy, were not captured. However, if the scales were to be clinically integrated, it is unlikely that contemporary survivors would undergo radiation therapy due to its associated side effects and the availability of more effective treatment alternatives [1]. Additionally, due to unclear instructions in the intake surveys, some of the sociodemographic data of young survivor participants could not be analyzed, as they pertained to their parents instead. The free-text question regarding treatment history also resulted in participants not mentioning enucleation, even if it was applicable. For future studies, the instructions will be better clarified with input from patient advocates, and free-text questions will be minimized. The age of the children discussed by parent participants was also not collected, making age-related appearance-based comparisons in survivors under 8 years of age not possible.

## Conclusion

In conclusion, with input from survivors, parents, clinicians, and scientists, the FACE-Q was assessed for its content validity and further modified. All modifications, including the development of the novel prosthetic-specific scale, demonstrate the necessity of conducting this essential step with patient input when adapting or developing PROMs. The next step in the development of the first validated PROM for retinoblastoma survivors, is to evaluate its psychometric properties through employment of Rasch analysis which is currently ongoing, as well as the cultural and linguistic adaptability of the PROM to ascertain validity across diverse populations. Following full validation, the adapted FACE-Q will fill a critical gap within retinoblastoma care and research, enabling patient input and standardized assessment of appearance-related QoL in clinical and research settings.

## Supporting information

S1 Text Interview guide for cognitive interviews. (PDF)

S2 Table Results from intake survey. (XLSX)

S3 Table Quantitative summary of modifications applied to FACE-Q scales and checklists. (XLSX)

S4 Table New concepts elicited. (XLSX)

S1 File Meeting Abstracts. (DOCX)
