# Supplementary material for: Content validity assessment and modification of the FACE-Q craniofacial module for retinoblastoma survivors
Source: PLoS One. 2026 Mar 26;21(3):e0339657. doi: 10.1371/journal.pone.0339657 (PMC13020849; doi:10.1371/journal.pone.0339657)
Supplement: S1 Text — (PDF) [file pone.0339657.s001.pdf]

Interview Guide

INTRODUCTION

Welcome Statement

Hello! My name is [Interviewer's Name] and I will be your interviewer for today. [Note-Taker's Name] is also here with us today to take notes on our discussion. We are both part of the research study team here at SickKids, and we want to start off by thanking you for taking the time out of your day to participate in our study! How are you?

Topic Overview/Goals

Great! So, I will start off by just briefly going over how things will go today. If you have any questions, I would be more than happy to answer them. Do you have any questions now?

[If yes, answer the questions.]

[If no] OK then. I will remind you that you are welcome to interrupt and ask me questions at any time during the interview, especially if you feel confused or do not understand something.

During today's interview, we will be reviewing some sections of a type of questionnaire called a patient-reported outcome measure, or PROM for short. These survey-type questionnaires are designed to help healthcare professionals understand your experiences with living with your condition and receiving treatments from your point of view.

[Round 1 Interviews: The PROM we will be looking at today is called "FACE-Q CRANIOFACIAL," or "FACE-Q" for short.]

[Round 2 Interviews: The PROM we will be looking at today is a modified version of a PROM called "FACE-Q CRANIOFACIAL," or "FACE-Q" for short. I will mention why it is the "modified version" in a few moments.]

Just as a little bit of background, FACE-Q has been tested in and developed with the input of over 2000 patients worldwide, all 8 to 29 years of age, and with conditions associated with visible and/or functional facial difference, or in other words, patients whose appearance, from the neck and above, has been affected by a condition/syndrome/illness.

FACE-Q has sections of questions that are related to the eye, but it has not been tested or validated in patients with eye conditions.

[For interviews with patients: For our study, we are interested in using the FACE-Q to develop a PROM specific to corneal anesthesia, retinoblastoma, and strabismus for patients who are 8 years old or older.]

[For interviews with parents/legal guardians: There is also no parent-proxy version of this PROM. This means that the patient experience and treatment outcomes have only been evaluated for patients aged 8 to 29 years old who are able to self-administer the PROM. The experience or treatment outcomes of younger patients, patients who are hard of hearing, or patients with developmental disabilities who cannot self-administer the PROM, however, have not been assessed. A parent-proxy version of the FACE-Q would be a version that would be administered to parents or legal guardians of patients under 8 years of age, or patients who are hard of hearing or with developmental disabilities, to evaluate their child's experience and treatment outcomes.

For our study, we are not only interested in using the FACE-Q to develop a PROM specific to corneal anesthesia, retinoblastoma, and strabismus patients for patients who are 8 years old or older, but to also use it to develop a parent-proxy version to evaluate the experience and outcomes of patients in these populations who are under 8 years of age, hard of hearing, or who have developmental disabilities.]

[Round 1 Interviews: To do this, today we will be looking at questions in scales related to the eye, appearance distress, school function, social function, and psychological function, and assess whether you think (1), the questions are easy to understand, (2) the questions are relevant to your/your child(ren)'s experience, (3) the existing questions need to be modified, and (4) there are additional or unnecessary questions that need to be added or removed. The reason we have decided to look at these scales is because previously, patients and their parents/legal guardians have identified appearance, vision function, and psychosocial outcomes to be important to them.]

[Round 2 Interviews: A few months ago, we tested questions in scales related to the eye, appearance distress, school function, social function, and psychological function on other survivor/parent/legal guardian participants like you. We asked the participants if the questions or scales needed to be modified to be easier to understand and to be more relevant to and comprehensive of their/their child's experience. Based on their feedback, we made some modifications to some of the FACE-Q questions. Today, we want to review these modified questions, along with some of the original questions that patients decided were relevant with you. As we evaluate each question, we will ask you whether you think the questions are (1) easy to understand, (2) relevant to your/your child(ren)'s experience, (3) need to be further modified, and (4) if there are additional or unnecessary questions that need to be added or removed. In other words, we want to receive your input to evaluate the acceptability and relevance of our modifications.]

To clarify, we are not using this PROM to collect data on you/you or your child(ren), but rather testing it to see if it is relevant to your/your child(ren)'s lived experiences and treatment outcomes. I also want to let you know that I did not write any of these questions, so do not worry about hurting my feelings if you criticize them – my job is to find out what is wrong with them, and then to work with my study team to improve them based on your feedback.

[Ask if they have any questions]

[If only audio recording: I would also like to remind you that this interview will be audio recorded. This is simply so that we can transcribe the recording later to analyze all information shared during our discussion. When we transcribe the data, your information will be de-identified, so everything you say today will remain confidential. Aside from members of the study team, no one will look at the data before it has been de-identified. Is this okay?]

[If audio and video recording: I would also like to remind you that this interview will be audio and video recorded. This is simply so that we can transcribe the recordings later to analyze all verbal and non-verbal data, meaning we will look at things that were said during the interview, as well as any facial expressions or movements that may be important. When we transcribe the data, your information will be de-identified, so everything you say today will remain confidential. Aside from members of the study team, no one will look at the data before it has been de-identified. Is this okay?]

Great! I would also like to mention that I am not a medical expert, and my role today is to review parts of the FACE-Q PROM with you and to gain an understanding of your experience with and opinions on the questions there. If you do have any medical-related questions, please bring them up with your/your child(ren)'s doctor and/or ophthalmologist at your/their next appointment.

Also, if at any point during the interview, you feel uncomfortable, distressed, that you do not want to talk about something, or if you simply need a break, please let me know. We will go at your pace.

[One more chance to ask questions]

Alright! We are now ready for your interview.

ROUND 1 COGNITIVE DEBRIEFING PROBES

Instructions:

[The interviewer will instruct the participant to read the instructions in their head, or out loud as per their level of comfort. Once the participant has finished reading the instructions, the interviewer will ask all of the following probes:]

1. In your own words, can you please tell me what the instructions are asking you to do?
2. Is this a good timeframe for this question?
3. Do you have any suggestions for making the instructions easier to understand?

After Each Item:

[The interviewer will instruct the participant to read each item in their head, or out loud as per their level of comfort. The interviewer will then ask the participant to think out loud by saying: "I would like you to think out loud as you think of which answer option to choose. This will help me understand how you interpret the question and be aware of what things you think about to help you choose your answer." Once the participant has finished thinking out loud and picks an answer, the interviewer will ask some or all of the following probes, depending on missing content from the participant's think-aloud process.]

1. In your own words, can you please tell me what this item, or question, is asking you?
2. Are there any words in the question that are difficult to understand? Do you find any words offensive? [If participant responds with "yes," follow up with "Do you have any suggestions to make this question more appropriate/easier to understand?"]
3. Do you think the response options are appropriate for this item, or question? [If participant responds with "no," follow up with "Do you have any suggestions on appropriate response options?"]
4. How did you arrive at your answer?
5. Was this item easy or hard to answer? [If participant responds with "hard," follow up with "Do you have any suggestions to make this question easier to answer?"]
6. Did you find the response options appropriate for this item? [If participant responds with "no," follow up with "Do you have any suggestions to make the response options a better fit for this question?"]

7. Does this item measure a concept that is important or relevant to you/your child(ren) and your/your child(ren)'s experiences? [If participant responds with "no," follow up with "Would you suggest removing or modifying this item from this scale and why?"]

After Each Scale:

[After reviewing all the items in a particular scale, the interviewer will ask all of the following probes:]

1. Are there any concepts, or ideas, that are important to you/your child(ren), and your/your child(ren)'s experience but were not mentioned here?
2. Is there anything else that you would like to comment on?

At the End of the Interview:

[After reviewing all of the scales chosen to be evaluated for this study, the interviewer will ask all of the following probes:]

1. What are your thoughts on the scales we went through today?
2. Is there anything that you feel is important to you/your child(ren), and your/your child(ren)'s experience, that wasn't covered today?*
3. Is there anything you would suggest changing about the scales we went through today?
4. Do you have any final comments or suggestions?

*Some of the scales that will be evaluated for this study (i.e., School Function) may not be relevant for older participants. In such cases, at the end of the interview (and as a follow-up to this scripted probe), probes regarding the relevance of topics that may be of importance and better suited towards the older participants as per the literature (i.e., Workplace Function, Work-Related Stress, Family Planning, Relationships, etc.) but not brought up by the participants themselves may be asked.

[In collaboration with the modification panel (see Protocol), additional probing questions tailored to test the acceptability and relevance of the modifications made by the research study team based off the first round of interviews will be added to this guide.]

CONCLUSION

And that is it! Thank you so much again for participating in this study and for providing us with your unique and valuable feedback.

I would like to let you know about a feedback form that we are sending to all participants by e-mail. [Verify their e-mail address with them] The feedback form is anonymous and voluntary. If you are interested however, we are requesting you to provide any feedback on your interview today and recommendations on how the interview can be improved. There will also be a section for you to add any additional comments or suggestions that you may think of later or that we were not able to discuss today.

If you have any questions after today, you can always feel free to contact Farheen Khan at farheen.khan@sickkids.ca. Just remember to not include any personal information in the e-mails since e-mails are not always very secure.

As a token of appreciation, and to express the research study team's gratitude, I have a Certificate of Participation for you. [If in person, hand the certificate to the participant. If virtual, ask if the participant would prefer to receive the certificate via e-mail, or have a physical copy mailed to them. Ensure to double check and record their e-mail address or mailing address – whichever is applicable.]

Thank you again. Take care and have a wonderful rest of the day/evening/weekend/week!

ROUND 2 COGNITIVE DEBRIEFING PROBES

OVERALL:

- Comment section per scale or overall?
- Do you prefer "eye(s)" and "eyelid(s)" or "eyes" and "eyelids"? Timeframe: month or week?
- Parent Participants: How can we clarify that parents should be thinking from THEIR CHILD'S perspective and not their own?

APPEARANCE OF THE EYES

- Is the qualifier about glasses helpful in the instructions: "If you wear glasses or a patch, please answer thinking of how you feel with your glasses or patch"?

- Is there a way we can better clarify "suit" in #1 (How much do you like how well your eye(s) suit(s) your face?)
- Parent Participants: "How does your child feel" vs. "How happy is your child with" question stem

APPEARANCE DISTRESS

- What do you think about changing #9 (I get upset when people stare at me) to "I don't like/dislike it when people look at or talk about me/my face?" Is "me/my face" better than "me" or would you prefer "me/my eyes"? Is qualifier enough - best to keep it general?
- Is the qualifier in the instructions (With your eye condition in mind) enough? Would you prefer to keep this section general or specific to the eye/eye condition? Should the instructions specify thinking of the face or the eyes?

PSYCHOLOGICAL FUNCTION

- Should this section be general or specific to the eye/eye condition?
- Do you find any redundancy between #4 (I feel okay about myself) and #9 (I feel great about myself)?
- Do you find redundancy between #5 (I believe in myself) and #6 (I am proud of myself) or #5 and #8 (I feel confident)?

SCHOOL FUNCTION

- For #8 (I feel safe at school (not bullied)), should "safety" and "bullying" be separated into two different questions?
- What do you think about adding the question, "I feel supported at school (e.g., resources)"to this section?

SOCIAL FUNCTION

- What do you think about adding "time you spent with others" in the instructions?
- What do you think about adding the questions "I feel confident when I am with people I know," "I feel confident when I am with new people," and "It's okay if people ask me questions about my eyes"? For the last question, would you prefer the word "eyes" or "face"?

EYE FUNCTION

- For each question, ask "has this ever happened to you/do you experience this now?"
- Explain why vision-related questions were removed.
- Do you like the right eye vs. left eye sub-division or would you prefer a living right eye vs. living left eye vs. prosthetic right eye vs. prosthetic left eye sub-division?
- In "My eye always moves like my other eye," is "other eye" appropriate wording?
- Is "living eye" the best term to use for the non-prosthetic eye?

EYE ADVERSE EFFECTS

- For each question, ask "has this ever happened to you/do you experience this now?"
- Do you like the living right eye vs. living left eye vs. prosthetic right eye vs. prosthetic left eye sub-division or would you prefer a right eye vs. left eye sub-division?
