# Supplementary material for: Content validity assessment and modification of the FACE-Q craniofacial module for retinoblastoma survivors
Source: PLoS One. 2026 Mar 26;21(3):e0339657. doi: 10.1371/journal.pone.0339657 (PMC13020849; doi:10.1371/journal.pone.0339657)
Supplement: S1 File — (DOCX) [file pone.0339657.s005.docx]

# Meeting Abstracts:

- - - 1. Evaluation and adaptation of the FACE-Q patient-reported outcome measure for retinoblastoma patients. International Society for Quality of Life Research Annual Meeting in Cologne, Germany, October 13-16, 2024; Qual Life Res. 2024;33(Suppl 1):1–235. <https://doi.org/10.1007/s11136-024-03786-x>

# Evaluation and Adaptation of the FACE-Q Patient-Reported Outcome Measure for Ophthalmology Patients. Association for Research in Vision and Ophthalmology (ARVO) Annual Meeting in New Orleans, LA, April 23-27, 2023; Invest. Ophthalmol. Vis. Sci. 2023;64(8):4230.

1. Evaluation and adaptation of the FACE-Q patient-reported outcome measure for retinoblastoma. 54th Congress of the International Society of Paediatric Oncology in Barcelona, SP, September 28-October 1, 2022; Pediatric Blood Cancer; 2022:69(e29952). https://doi.org/10.1002/pbc.29952.

**Submitted as:** Research Article to PLOS ONE
